# “Sometimes It's Difficult to Have a Normal Life”: Results from a Qualitative Study Exploring Caregiver Burden in Schizophrenia

**DOI:** 10.1155/2014/368215

**Published:** 2014-04-03

**Authors:** Adam Gater, Diana Rofail, Chloe Tolley, Chris Marshall, Linda Abetz-Webb, Steven H. Zarit, Carmen Galani Berardo

**Affiliations:** ^1^Adelphi Mill, Grimshaw Lane, Bollington, Cheshire SK10 5JB, UK; ^2^Roche Products Ltd., Hexagon Place, 6 Falcon Way, Welwyn Garden City, Hertfordshire AL7 1TW, UK; ^3^305 Health & Human Development East, The Pennsylvania State University, University Park, PA 16802, USA; ^4^F. Hoffmann La-Roche Ltd., Grenzacherstrasse 124, 4070 Basel, Switzerland

## Abstract

*Objectives*. As a disease typified by early onset and chronic disease course, caring for a person with schizophrenia may have a significant impact on caregivers' lives. This study aimed to investigate the subjective experiences of caregivers of people with schizophrenia as a means of understanding “caregiver burden” in this population. *Methods*. Face-to-face qualitative interviews were conducted with a diverse sample of 19 US-English speaking caregivers of people with schizophrenia (who were at least moderately ill). Interview transcripts were analyzed using grounded theory methods and findings used to inform the development of a preliminary conceptual model outlining caregivers' experiences. *Results*. Findings support assertions that people with schizophrenia were largely dependent upon caregivers for the provision of care and caregivers subsequently reported lacking time for themselves and their other responsibilities (e.g., family and work). Caregiver burden frequently manifested as detriments in physical (e.g., fatigue, sickness) and emotional well-being (e.g., depression and anxiety). *Conclusions*. Caring for a person with schizophrenia has a significant impact on the lives of informal (unpaid) caregivers and alleviating caregiver burden is critical for managing individual and societal costs. Future research should concentrate on establishing reliable and valid means of assessing burden among caregivers of persons with schizophrenia to inform the development and evaluation of interventions for reducing this burden.

## 1. Introduction

Over the past 50 years, as evidenced by the closure of mental hospitals and advent of community-based care, there has been transition of care for schizophrenia patients from formal hospital-based healthcare systems to outpatient and community services. The financial burden (in terms of direct and indirect costs) of community-dwelling patients with schizophrenia is high with estimated annual costs in the US of $23 billion [1].

It is estimated that 50–90% of people with chronic psychiatric illness live with their families or friends [2, 3] and informal caregivers (i.e.,* “a person who has significant responsibility for managing the well-being of a person diagnosed with schizophrenia in an unpaid capacity”*) [4] provide an important service by reducing need for formal care and burden upon healthcare systems [5]. This is true not just of schizophrenia but also of other medical conditions such as stroke [6], cancer [7] and dementia [8], with estimates in 2011 suggesting that the economic value of all informal caregivers' unpaid contributions in the US was $450 billion, having increased from $375 billion in 2007 [9, 10].

Caregiving responsibilities can place a burden on caregivers' daily lives, physical health, and emotional well-being [11–37]. High levels of caregiver burden can have considerable consequences for patients and wider society as a whole as well as for caregivers themselves. For instance, should the impact on the caregiver become too great, then there could be consequences on the delivery of care to patients, an increase in unmet needs of overburdened caregivers [38], and an increasing reliance on formal, paid assistance [39, 40] resulting in costs to healthcare systems and wider society. In addition, caregiving has significant impact on informal caregivers' own physical and psychological health [13]. Alleviating caregiver burden, therefore, is now recognized as a key outcome for healthcare interventions (pharmacological and psychosocial) designed to manage schizophrenia.

Caregiver burden is a complex construct with two distinct components: “objective burden” which refers to the tangible impact of caregiving on a caregiver's life (e.g., financial loss and time lost from daily activities) and “subjective burden” which refers to the caregiver's perception of burden of care [39]. Objective burden may be assessed by a variety of means but subjective burden can only be known by caregivers themselves and it is important that such reports come directly from caregivers themselves. In order to monitor caregiver burden, there is therefore a need for accurate and reliable self-report measures of the impact of caring for a person with schizophrenia. These measures would be important in understanding the impact on caregivers, identifying those caregivers at risk of significant health, social, and financial impacts, and evaluating the efficacy of interventions designed to reduce, alleviate, or protect against caregiver burden.

Although existing measures of caregiver burden have previously been used in schizophrenia, there is little evidence that the content validity of these measures in this population has been confirmed in line with well-established guidelines for the development and evaluation of self-report measures [41]. Consistent with these guidelines, there is a need to understand representation of caregiver burden from caregivers themselves. Although previous research has highlighted the impact of caring for a person with schizophrenia on all aspects of a caregiver's life [42], no studies have thus far developed a conceptual model, which defines the concepts of interest [43, 44], outlining the subjective experiences of caring for a person with schizophrenia. In addition, prior research has identified a number of factors which may impact the subjective burden experienced by caregivers. For example, there is evidence to suggest that

(i) specific clusters of schizophrenia symptoms may have a differential impact on caregiver burden [11, 45, 46] and high levels of symptomology have a significant impact on caregiver burden [47–50];
(ii) there are differences in the concept of caregiver burden in terms of ethnicity (with white caregivers more likely to report high levels of caregiver burden than African Americans) [42, 51, 52];
(iii) the gender of the person receiving the care (males with schizophrenia are more frequently associated with high levels of caregiver burden) [47, 53] and the person providing the care (female caregivers experiencing higher levels of burden than male caregivers) [11, 54] can influence caregiver burden;
(iv) the caregiver relationship to the person with schizophrenia (e.g., mother-daughter and father-daughter) and living situation (living with person or not) can affect the degree of caregiver burden [55];
(v) age of the person receiving care [56, 57] and age of the caregiver [58] may both have an independent effect on reported rates of caregiver burden (studies suggesting that younger people with schizophrenia and older caregivers experience higher levels of burden);
(vi) educational attainment may predict levels of caregiver burden, with those of lower educational backgrounds experiencing higher levels of caregiver burden [59].

The aims of the present study are to investigate, via qualitative interviews, the subjective experiences of informal caregivers of people with schizophrenia and to develop a preliminary conceptual model outlining the impact of caregiving on their daily lives.

## 2. Methods

### 2.1. Study Design

Face-to-face, open-ended, semi-structured, qualitative interviews were performed with 19 English speaking informal caregivers of people with a clinician confirmed diagnosis of schizophrenia in the US. Caregivers were identified for inclusion following referrals from patients of private clinicians. Clinicians first identified people with schizophrenia and determined their eligibility for participation in the study.

People with schizophrenia were aged 18 and above and were required to have been diagnosed according to the Diagnostic and Statistical Manual of Mental Disorders, IV, Third Revision (DSM-IV-TR) criteria, to be at least moderately ill (as defined by a Clinical Global Impression of Severity (CGI-S) score ≥4) and currently to be receiving a marketed atypical or typical antipsychotic. To be eligible for participation, people with schizophrenia were also required to be in receipt of care from an informal caregiver. People with schizophrenia were excluded if they had any other DSM-IV-TR Axis I diagnoses, alcohol, or substance dependence within 12 months or abuse within 3 months (with the exception of nicotine) or if they had dementia, delirium, or other amnestic disorder per DSM-IV-TR. They were also excluded if they had a diagnosis of mental retardation/severe organic brain syndromes/a prior or current general medical condition that may be impairing cognition or other neuropsychiatric functioning.

Once eligibility had been confirmed, the informal caregivers of people with schizophrenia were approached to take part in the study. To be eligible for participation, the caregivers had to be aged at least 18 years of age and currently providing care to the person with schizophrenia for at least 4 hours per week. Caregivers themselves were excluded from the study if they were judged by the recruiting physician to have severe neurological or cognitive deficits or an uncontrolled psychiatric condition that might affect their ability to participate in the interview.

Participants were recruited from three sites in the US to avoid geographical bias: Philadelphia (eastern US), Chicago (central US), and New Orleans (southern US). To ensure recruitment of a representative sample of schizophrenia caregivers, a series of recruitment targets were employed including the following.

(i) Schizophrenia subtype: at least two caregivers of people with each DSM-IV schizophrenia subtype.
(ii) Schizophrenia severity: at least five caregivers of people with schizophrenia that have CGI-S scores of 4, 5, and 6.
(iii) Gender: at least four males and at least four female caregivers of people with schizophrenia.
(iv) Relationship to person with schizophrenia: By gender: at least six gender-matched (e.g., daughter-mother, sister-sister) caregivers and at least six gender mismatched (e.g., daughter-father, husband-wife, son-mother, siblings, friends) caregivers. By living situation: at least 6 family members living with person with schizophrenia.
(v) Age: at least six caregivers under 60 years old and at least six caregivers 60 years and older.
(vi) Education: at least 20% of caregivers with high school as their highest level of education.

### 2.2. Ethics

The study was conducted in accordance with the Declaration of Helsinki and was approved by Copernicus Group, a centralized Institutional Review Board (IRB) in the US (IRB number: ADE2-12-035). Study procedures ensured that written informed consent was obtained from both people with schizophrenia and their caregivers prior to the collection of any data. Any data collected was anonymously stored in a computer database, maintaining confidentiality in accordance with national data legislation.

### 2.3. Interview Procedure

Each participant took part in a single one-hour interview. All interviews were conducted by a trained interviewer using a semistructured interview guide. This was informed by consideration of existing literature concerning the impact of caring for someone with schizophrenia or other mental health conditions. Initial questions were open-ended and designed to elicit spontaneous discussion concerning each caregiver's experience of providing care to someone with schizophrenia (e.g., “Tell me about your experience of caring for a person with schizophrenia”). Following these broad questions, more direct questions were used to elicit feedback on particular topics of interest, as identified within the existing research literature (e.g., “In what ways does caring for a person with schizophrenia affect you emotionally?”). Throughout this process, care was taken to avoid the use of leading questions, in order to ensure that caregivers' responses were reflective of their experiences and free from external bias. An enabling exercise was also used to facilitate spontaneous reporting of concepts whereby caregivers were asked to put together a collage representing their experiences of caregiving which they would take to the interview and discuss with the interviewer.

### 2.4. Analysis

All interviews were audio-taped and transcribed verbatim for the purpose of qualitative analysis. Written interview transcripts were then entered into a qualitative software package (Atlas. Ti) [60] which was used to facilitate the analysis of interview content.

Transcripts were analyzed using a grounded theory approach whereby sections of transcripts from individual participants (i.e., quotes) were assigned codes reflective of underlying concepts [61, 62]. In this approach, the meanings of concepts are discovered via the words and actions of participants from the ground up, rather than from application of a priori theory or understanding [63]. This is particularly useful where, as here, the intention is to build an overall picture of participants' experiences to inform development of a preliminary conceptual model. Analysis was performed by two primary researchers and the code list, which was driven by patient quotes, was regularly reviewed and agreed among the wider research team.

There exist no definitive guidelines regarding recommended sample sizes for qualitative studies and available guidance actually states that the number of participants is not as critical as interview quality, with sample size depending on the completeness of information obtained from the analysis of interview transcripts [41]. That the concepts elicited by caregivers had been fully explored during the interviews was assessed by confirmation of conceptual saturation, defined as the point at which no new relevant or important information emerges with the collection of more data [41, 61, 64]. During analysis, spontaneously reported concepts were therefore differentiated from those which caregivers discussed when prompted, allowing for assessment of conceptual saturation. Recent investigations suggest conceptual saturation is typically achieved in sample sizes of 12-13 [64, 65]. Although the primary purpose of qualitative research is not to assess concept frequency, a count of the number of caregivers who mentioned a given concept at least once during an interview was recorded during the process of analysis in order to provide an indicator of the relative importance of each concept within the study sample.

## 3. Results

### 3.1. Demographic and Clinical Characteristics

Although it is recognized that future qualitative research involving people with schizophrenia may increasingly focus on positive and negative symptoms as a means of differentiation, the current study ensured that a diverse sample of people with schizophrenia and their caregivers was recruited. The average age of the people with schizophrenia was 51.8 years (21–82) and 12 (63%) of the sample were females. People with schizophrenia had been diagnosed for an average of 16 years and were diagnosed with paranoid (*n* = 10, 53%), disorganized (*n* = 4, 21%), undifferentiated (*n* = 2, 11%), catatonic (*n* = 2, 11%), and paranoid plus disorganized (*n* = 1, 5%) DSM-IV-TR subtypes of schizophrenia. People had varying severities of schizophrenia but as per the inclusion criteria were at least moderately ill, with 14 people with schizophrenia (74%) having a CGI-S score of 4-5 and 5 (26%) having a CGI-S score of 6-7 [47–50].

The average age of caregivers was 51.6 years and caregivers were predominantly female (79%), which is consistent with previous research in samples of people with schizophrenia [24, 66]. The sample of caregivers included representation of Black/African American, White, and Hispanic/Latino caregivers and, of the total sample, 63% of the caregiver-person with schizophrenia dyads were gender matched. Summaries of the demographic characteristics for the people with schizophrenia and their caregivers recruited into the study are provided in Tables 1 and 2 respectively. Relevant clinical characteristics for the people with schizophrenia are also included in Table 1.

### 3.2. Enabling Exercise

**Figure 1:**
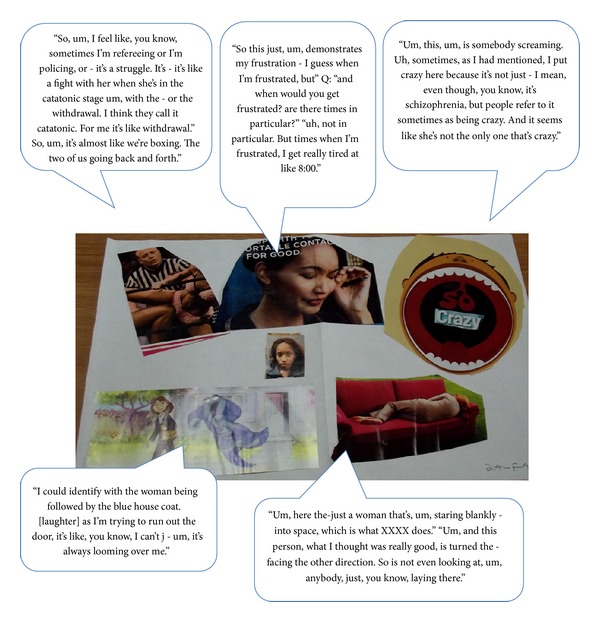
Caregiver ID: 214-F-48-C.

**Figure 2:**
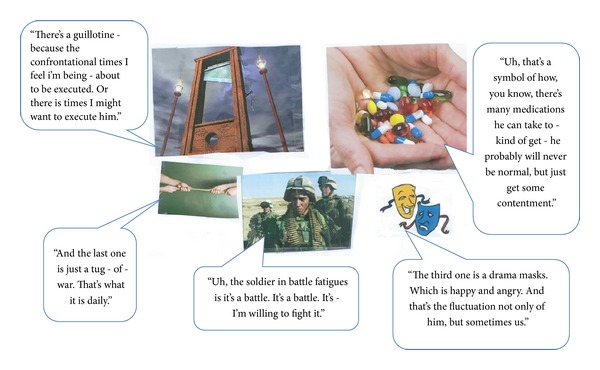
Caregiver ID: 102-M-40-C.

**Figure 3:**
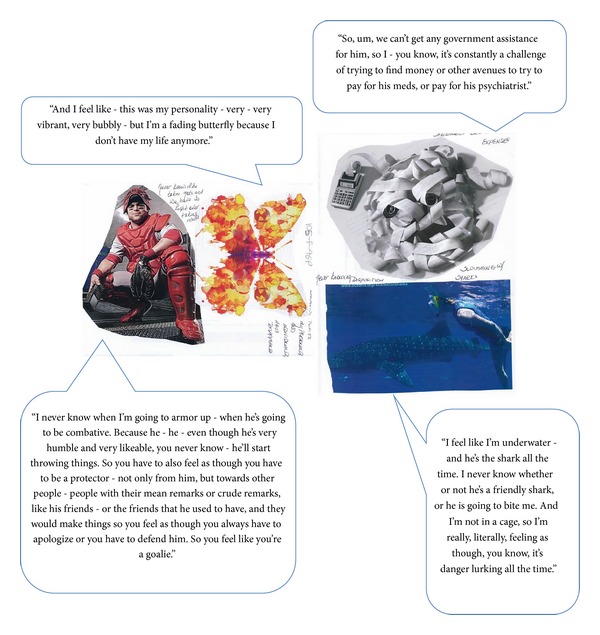
Caregiver ID: 105-F-46-P.

Creative collage tasks can be useful enabling exercises to encourage elicitation of relevant concepts from participants in qualitative research [67]. Prior to the interview, caregivers were asked to create a collage representing their experiences of caring for a person with schizophrenia, which was to be used to facilitate discussions during the interview. All 19 caregivers completed this exercise. Individual collages all made reference to multiple concepts, many of which are discussed below. Select examples of collages prepared by caregivers alongside feedback from caregivers during the interview are provided in Figures 1, 2, and 3.

### 3.3. The Experience of Caring for a Person with Schizophrenia

#### 3.3.1. Summary of Interview Findings

Findings from interviews with caregivers in this study suggest that “caregiver burden” can be understood in terms of 6 key dimensions: emotional well-being, caregiver concerns, physical impact, impact on daily activities, financial demands, and impact on relationships. Furthermore, study participants also reported that caring for a person with schizophrenia can have a positive impact on their lives.

**Figure 4:**
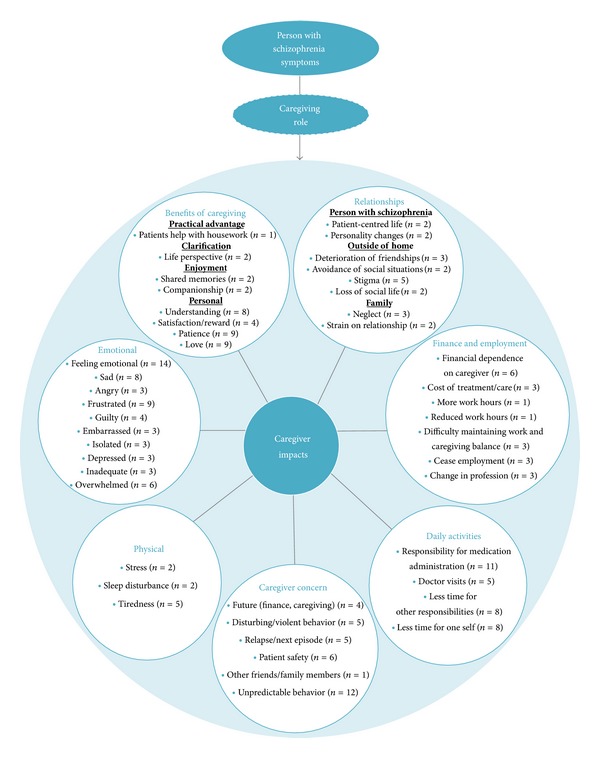
Preliminary conceptual model of caregiver experiences of providing care for a person with schizophrenia. Note: numbers refer to the number of caregivers who discussed each concept and for whom this concept was relevant. This preliminary conceptual model does not seek to convey the strength, direction, or nature of relationships between concepts and domains. This is to be the subject of future research.

Based on feedback from caregivers, a preliminary conceptual model was developed to outline and visualize the experiences of caregivers of people with schizophrenia, see Figure 4.

To confirm conceptual saturation in this population, a process of comparative analysis was used, in which concepts emerging from interviews 1–7 (Round 1) were compared to those emerging from interviews 8–13 (Round 2) and then 14–19 (Round 3). Very little new information emerged as data collection neared completion; 32/34 concepts (94%) relating to caregiver burden were elicited during the first 7 interviews, with only one new concept elicited during both Round 2 and Round 3 (financial dependence on caregiver). Findings therefore suggest that conceptual saturation in the present sample was achieved. The concepts most frequently reported by participants concerned the impact of caregiving on the caregivers' emotional state (i.e., feeling “emotional”, *n* = 14), the inconsistent and unpredictable nature of the person's schizophrenia (*n* = 12), the negative impact of caregiving on caregivers' relationships with other family members and friends (*n* = 11), and the impact of caregiving on caregivers' ability to work (*n* = 10).

#### 3.3.2. Emotional Impact

The most frequently reported impact of caring for a person with schizophrenia was feeling “emotional” which was reported by 14 (74%) of the caregivers. In addition, caregivers reported the specific emotions they experienced as a result of their role and incorporated a number of these into the enabling exercise. Caring for a person with schizophrenia was described as demanding by caregivers, leading them to feel overwhelmed (*n* = 6). Caregivers also reported that the role made them feel sad (*n* = 8). Feelings of sadness related to the caregivers themselves, the person with schizophrenia, or the feeling that they could not help the person with schizophrenia (e.g., by convincing them that delusions were not real). Caregivers said that some emotions were experienced as a result of the person with schizophrenia's behavior; these included frustration (*n* = 9), embarrassment (*n* = 3), anger (*n* = 3), and stress (*n* = 2).

The emotions experienced by caregivers were often changeable and unpredictable* “Emotionally, it's a rollercoaster”* (318-M-55-P), and when discussed in further details, caregivers reported that the unpredictable nature of the person with schizophrenia's behavior was often the reason for them feeling stressed, anxious, and worried (*n* = 12). Caregivers often used analogies to describe their changeable emotions, sometimes as part of their enabling exercise:* “White-water rafting. It's like - that's how I feel. Like, I'm going up and down with the whole deal. Yeah. You know, like bumps”* (106-F-52-D). Caregivers also used analogies to describe the unpredictability of the person with schizophrenia's behavior:* “I feel like I'm underwater - and he's the shark all the time. I never know whether or not he's a friendly shark, or he is going to bite me. And I'm not in a cage, so I'm really, literally, feeling as though, you know, its danger lurking all the time.”* (105-F-46-P).

#### 3.3.3. Caregiver Concerns

Caregivers reported a number of specific concerns relating to their role; they were afraid of what the future holds in terms of the person with schizophrenia's care (*n* = 4), worried about the person with schizophrenia having an “episode” (*n* = 5), and worried that the person with schizophrenia's symptoms would get worse (*n* = 3).

#### 3.3.4. Impact on Daily Activities

The main impact of caring for a person with schizophrenia on participants' daily activities was the lack of time, both for themselves (*n* = 8) and for their other responsibilities (*n* = 8):* “Um, I do not have much of a life anymore. So between running around for the kids and caring for my mother, there's not much time for anything else.”* (103-F-38-P). Caregivers spoke about having to cancel plans (*n* = 1) and not being able to spend time with other family members (*n* = 4). They also felt that they had lost control of their lives which now centered around the person with schizophrenia (*n* = 2):* “In the know means you have to always be - have knowledge about the situation - the medication - her life, where she is going, what doctor appointments. You cannot just - just forget about anything.” *(104-F-48-D).

Caregivers felt that people with schizophrenia were dependent on them for help with numerous aspects of their lives (*n* = 5). They were relied upon to take the person with schizophrenia to appointments (*n* = 3), remind the person with schizophrenia about their personal hygiene (*n* = 2), take medication (*n* = 6), and even to eat (*n* = 1). They found their role difficult (*n* = 3) and felt uncertain about the best way to fulfill it (*n* = 3). One caregiver said that he felt he was not able to care for the person with schizophrenia as well as he would have liked:* “So the challenge is we're not specific in that aspect. It's myself, lady friend - sometimes my son, brother. There's a neighbor. But there is not that specificity, you know, therapist on - on, uh, site. People that can deal with it. Uh, even maintenance people.”* (102-M-40-C). Many caregivers included their caregiving responsibilities into the enabling exercise, such as ensuring the person with schizophrenia took their medicine (*n* = 9) and having to take them to the doctors (*n* = 2).

Despite caregivers often having large families, they sometimes felt alone in caring for the person with schizophrenia (*n* = 3) and other family members and friends would not help out as much as the caregiver would have liked:* “I'm the only one that - that - that does it now. That actually cares. Because of - because of that disease - it has pushed my siblings, relatives away because they just do not know how to handle it, per se.”* (318-M-55-P).

#### 3.3.5. Physical Impact

Caring for a person with schizophrenia affected caregivers' physical health (*n* = 9) as it left them feeling tired (*n* = 5), worn out (*n* = 2), drained (*n* = 3), and stressed (*n* = 2). Interestingly, the only physical impacts to emerge from the enabling exercise were tiredness and stress. In some cases the additional burden of being a caregiver exacerbated their existing problems:* “Um, so yeah, it did affect me physically. And I remember one time the doctor, the gastroenterologist, said to me if you do not divorce him, your stomach will never be OK.” *(109-F-69-P). In addition to this, some caregivers talked about disturbed sleep due to the person with schizophrenia's behavior and having to continue their caregiving role through the night (*n* = 2).* “If she's already in an episode - I might not be able to sleep thoroughly. I might have to stay up with her for hours - on end at times.”* (212-M-39-P).

Caregivers also felt that the emotional impacts of worry, anxiety, and stress affected them physically (*n* = 3). The physical demands of caregiving meant one caregiver felt she wouldn't be able to continue caring for the person with schizophrenia for much longer.

#### 3.3.6. Financial Impact/Impact on Employment

The person with schizophrenia was often described as being dependent on the caregiver for financial support (*n* = 6) and one of the main financial demands for caregivers was the cost of medication (*n* = 3).* “Well, financially, because, um, sometimes we need people to, uh, to stay with her, to watch her, you know, medical bills for certain things at times.” *(212-M-39-P).

As a result of this financial dependence, some caregivers said that they did not have enough money to care for the person with schizophrenia in addition to the rest of their expenses (*n* = 6). They saw the money spent on caregiving as a sacrifice (*n* = 1) and were spending their own savings (*n* = 2). A contributing factor to the financial difficulties faced by caregivers was the impact of their role on their work (*n* = 10). Caregivers either had to give up work (*n* = 3), change the way that they worked, or change their profession (*n* = 3). Of those caregivers that remained at work, their role still interfered with their working life as they would get disturbed at work by the person with schizophrenia, or suffered from a lack of sleep (*n* = 3). For caregivers who had given up work, it sometimes meant that others had to increase their work load to compensate (*n* = 1).* “I cannot go to work. So financially, it has put a burden. My husband had to take a - second job because - to - to supplement my income and it still is not supplementing it.” *(103-F-38-P).

#### 3.3.7. Impact on Relationships with Others

The caregivers' relationship with the person with schizophrenia often had an impact on their relationships with other family members and friends in a negative way (*n* = 11). Caregivers saw less of their children than they would have liked (*n* = 3) and noticed strain in their marriage (*n* = 2).* “It affects me where I do not have as much time. Like, my - both of my kids are athletes, so it's not like I have as much time to do their games.” *(106-F-52-D).

Outside of family life, caregiver social life was also affected (*n* = 7); caregivers had to give up their previous social life (*n* = 2) and felt they had to stay in or near the house (*n* = 2). In addition to this, they felt uncomfortable about having friends over to see them (*n* = 2) because they did not want their friends to witness the person with schizophrenia's behavior.* “Um, well, it's kind of affected my social life a lot. Um, I do not have friends that come over too much anymore since she's been living with me now.”* (211-F-59-D).

#### 3.3.8. Impact on Relationship with Person with Schizophrenia

In the enabling exercise, some caregivers described their role as a fight or battle with the person with schizophrenia (*n* = 4):* “I mean, it's just a battle sometimes to get her to get dressed, take a shower, take her medicine, to eat. It's just a battle.” *(211-F-59-D). However others said that they worked with the person with schizophrenia as a team (*n* = 3):* “I chose this one because it takes teamwork to - to climb this mountain. And without teamwork, you know, they - they - they - they wouldn't have been successful. So that's what I find, that he and I, we are a team. We are a team.” *(315-F-59-D). Caregivers also described trying to understand what the person with schizophrenia is going through as a puzzle or maze (*n* = 2):* “I try to s - you know, try to feel what she's feeling. So - and you know, if I cannot - I cannot understand it, I cannot get it, it's a maze to me.”* (212-M-39-P).

#### 3.3.9. Positive Impacts

Despite the high levels of burden associated with caring for a person with schizophrenia, 17 caregivers also spoke about the positive aspects of their role. The most commonly reported benefit was the love that the caregiver shared with the person with schizophrenia (*n* = 9):* “Right. And then I have a heart because I love her. And I probably love her even more now.”* (104-F-48-D).

Other benefits included being able to care for the person with schizophrenia and notice problems (*n* = 3), ensuring the person with schizophrenia was safe and being cared for (*n* = 3), and understanding what the person with schizophrenia was going through (*n* = 6). Caregivers also felt they had a higher level of patience (*n* = 9), were a “better person” (*n* = 1), had grown as a person (*n* = 2), and felt reward and satisfaction (*n* = 4):* “Uh, the benefits? Patience. The patience and the love you have you know. Those are the most important.”* (108-M-50-D);* “- but I think, for me, like I said, it's - it's helped me develop more as a person.”* (213-F-38-P).

Some caregivers also said that they enjoyed having the person with schizophrenia at home, had fun with them, and saw them as a companion.

## 4. Discussion

Caring for a person with schizophrenia was found to have significant implications for caregivers' perceived physical and emotional well-being and these findings are consistent with those from prior qualitative investigations in informal caregivers of schizophrenia patients [14–37]. Thus, findings from the current study serve to highlight the burden and unmet needs among caregivers of persons with schizophrenia.

The preliminary conceptual model which was developed to outline the experiences of caregivers of people with schizophrenia, as informed by qualitative findings, demonstrates that the caregiving role has a significant impact on numerous facets of caregivers' lives. In addition to identifying potential measurement concepts, such models may also inform decisions regarding how best to measure these concepts in research studies and help to contextualise findings from studies in this area [43]. A limitation of this preliminary model is that, in seeking to provide a rich and comprehensive understanding of the concepts of relevance to caregivers of people with schizophrenia using qualitative evidence, we were not able to confirm the influence of moderating factors and draw generalizable conclusions regarding interrelationships between concepts. Qualitative research is often used in psychiatry to provide a broader and more thorough understanding of the patient or caregiver experience; however, statistically confirmed, quantitative methods are required to investigate concepts and their interrelationships in more details [68]. Future research, including larger quantitative studies, may be used to validate the model and could provide further insight into how concepts relate to each other and how moderating variables affect the strength and direction of these relationships.

A diverse sample of caregivers of people with schizophrenia was interviewed as part of this study. This included caregivers of people with different subtypes of schizophrenia as determined by the DSM-IV. While this subtype classification has since been discontinued in the DSM-V, this criterion (alongside targets for clinically defined severity) offers assurances that the views of caregivers of a clinically diverse population were considered.

### 4.1. Implications

These findings reiterate the need for strategies to reduce caregiver burden in informal caregivers of persons with schizophrenia. This is in accordance with recent health initiatives, which highlight the need to prioritize the mitigation of caregiver burden and implement strategies into real-world practice (e.g., Centers for Disease Control and Prevention) [69].

Feedback from study participants indicates that caregiver burden may vary according to certain caregiver and patient characteristics; for example, feedback from participants suggested that the concept of caregiver “burden” was less relevant among Black-African American caregivers than among White caregivers. This appeared to be largely due to a strong focus on faith and religion:* “You have to take these things. If you have a belief in God, you have to take that system - and employ it in this situation… Whatever God you serve” *(318-M-55-P). Similarly, differences according to the age of the caregiver and relationship to the person with schizophrenia were noted. Such findings are consistent with previous investigations among schizophrenia caregivers which found that a number of external factors contribute to the level of caregiver burden experienced and therefore highlight the potential need for individually tailored interventions [11, 42, 45–53, 59, 70]. Findings, however, should be interpreted with caution due to the small sample sizes in the current study. Further exploration of differences among subsets of caregivers of people with schizophrenia within larger quantitative studies should be an avenue for future research.

These findings highlight the need for assessment of the impact on caregivers, in addition to patient-centered outcomes, in both clinical practice and clinical research. In clinical practice, routine assessment of impact on caregivers could serve to identify those caregivers most at risk of ill health as a result of providing care for a person with schizophrenia. Such information could be used to inform of the development and evaluation of individually tailored solutions designed to alleviate caregiver burden. This study suggests that caregiver burden is closely linked to symptom manifestation in the person with schizophrenia: Q:* “- are there any that impact you more or less than others?”* 109-F-69-P:* “Yeah. When he gets abusive to me.”* Assessments of caregiver burden as an exploratory endpoint in clinical trials could therefore potentially serve as an additional means of evaluating improvement in person with schizophrenia functionality (e.g., independence) and the development and evaluation of interventions (e.g., support networks).

Although such outcomes are unlikely to support product labeling claims and indications for novel pharmacological therapies, this information is important for communicating the wider benefit of treatments to patients/caregivers, payers, and prescribers. Health Technology Assessment (HTA) agencies increasingly require that patient relevant endpoints such as global and functional outcomes (readiness for discharge and readiness for work), quality of life, patient satisfaction, family burden, and resource utilization should be included in schizophrenia trials. The National Institute for Health and Care Excellence (NICE) HTA Guidance for schizophrenia, for example [71], emphasizes the need for assessment of caregiver burden and provision of adequate support to caregivers; highlighting that information regarding caregiver burden potentially has an important role to play in pricing and reimbursement decisions. Caregiver burden, therefore, may serve as a key indicator of treatment value, particularly in markets (e.g., UK) where decisions must be made regarding the most efficient allocation of finite healthcare resources.

#### 4.1.1. Current Challenges Associated with the Assessment of Caregiver Burden

There are a number of self-report instruments available to measure caregiver burden; however, none has been evaluated in accordance with current guidance for self-report measures [72]. It is important that any instrument assessing burden among caregivers of persons with schizophrenia assesses those particular issues of relevance and importance to such caregivers and demonstrates reliability and validity of assessment of caregiver burden in this population. Combined with existing qualitative research, findings from the current study could serve to inform modifications to existing measures or development of new measures of caregiver burden in persons with schizophrenia. Assessing quality of life in caregivers of people with schizophrenia could also inform assessment of utility which may have a direct influence of the cost-effectiveness analysis for schizophrenia interventions. This is increasingly important as HTA agencies seek to consider information concerning societal burden when making decisions regarding access to new therapies.

## 5. Conclusions

Caring for a person with schizophrenia places significant impact on caregivers' lives, manifesting emotionally, physically, financially, in daily activities, and in relationships. This burden may vary according to numerous individual factors. Findings highlight the need for valid and reliable means of assessing burden among caregivers of persons with schizophrenia so as to inform of the development and evaluation of interventions (e.g., support networks) for reducing caregiver burden.

## Figures and Tables

**Table 1:** Persons with schizophrenia and demographic and clinical characteristics (*n* = 19).

Demographic characteristic	Person with schizophrenia (*n* = 19)
Age	
Mean (range)	51.84 (21–82)
Gender *n* (%)	
Male	7 (37)
Female	12 (63)
Ethnicity *n* (%)	
Black/African American	12 (63)
Hispanic/Spanish American/Latin (of any race)	1 (5)
White/Caucasian	6 (32)
Years since diagnosis	
Mean (range)	16 (3–42)
Schizophrenia subtype *n* (%)	
Paranoid	10 (53)
Disorganised	4 (21)
Undifferentiated	2 (11)
Catatonic	2 (11)
Paranoid, disorganized	1 (5)
Schizophrenia severity *n* (%)	
CGI-S score 4	9 (47)
CGI-S score 5	5 (26)
CGI-S score 6	5 (26)
Medication *n* (%)	
Typical antipsychotics	
Haloperidol	5 (26)
Chlorpromazine	3 (16)
Atypical antipsychotics	11 (58)
Aripiprazole	4 (21)
Risperidone	5 (26)
Olanzapine	3 (16)
Quetiapine	0 (0)
Olanzapine/fluoxetine	1 (5)
Ziprasidone	2 (11)
Other	6 (32)
People with schizophrenia on both typical and atypical treatments	5 (26)

**Table 2:** Caregiver demographic characteristics (*n* = 19).

Demographic characteristic	Caregivers (*n* = 19)
Age	
Mean (range)	51.63 (28–69)
Gender *n* (%)	
Male	4 (21)
Female	15 (79)
Ethnicity *n* (%)	
Black/African American	11 (58)
Hispanic/Spanish American/Latin (of any race)	2 (11)
White/Caucasian	6 (32)
Relationship with person with schizophrenia *n* (%)	
Parent	6 (32)
Partner/spouse	2 (11)
Sibling	6 (32)
Son/daughter	3 (16)
Other (nephew, aunt)	2 (11)
Education *n* (%)	
High school diploma	7 (37)
College or university degree	5 (26)
Graduate or professional degree	5 (26)
Some years of college	2 (11)
Work status *n* (%)	
Working full or part time	12 (63)
Full time homemaker	3 (16)
Not working	1 (5)
Retired	2 (11)
Other	1 (5)
Coresidence *n* (%)	
Yes	15 (78.9%)
No	4 (21.1%)
Years spent caring for person with schizophrenia *n* (%)	
Mean (range)	10.24 (1–32)
Hours per week spent caring for person with schizophrenia *n* (%)	
<20	1 (5)
21–40	7 (37)
40+	11 (58)
Range	14–168
Mean	86.33
Gender match *n* (%)	
Yes	
Female	10 (53)
Male	2 (11)
No	7 (37)
